# Palladium‐Doped Cs_2_AgBiBr_6_ with 1300 nm Near‐Infrared Photoresponse

**DOI:** 10.1002/smll.202404188

**Published:** 2024-09-20

**Authors:** Hongwei Lei, Utkarsh Singh, Fuxiang Ji, Tinghao Lin, Libor Kobera, Yuequn Shang, Xinyi Cai, Weihua Ning, Andrii Mahun, Sabina Abbrent, Zuojun Tan, Jiri Brus, Dehui Li, Sergei I. Simak, Igor A. Abrikosov, Feng Gao

**Affiliations:** ^1^ College of Engineering Huazhong Agricultural University Wuhan 430070 China; ^2^ Department of Physics, Chemistry and Biology (IFM) Linköping University Linköping SE‐581 83 Sweden; ^3^ School of Optical and Electronic Information and Wuhan National Laboratory for Optoelectronics Huazhong University of Science and Technology Wuhan 430074 China; ^4^ Institute of Macromolecular Chemistry of the Czech Academy of Sciences Heyrovskeho nam. 2 Prague 162 00 Czech Republic; ^5^ Department of Physics and Astronomy Uppsala University Uppsala SE‐751 20 Sweden

**Keywords:** Cs_2_AgBiBr_6_, double perovskites, NIR light response, Pd doping, sub‐bandgap

## Abstract

Lead‐free halide double perovskite (HDP) Cs_2_AgBiBr_6_ has set a benchmark for research in HDP photoelectric applications due to its attractive optoelectronic properties. However, its narrow absorption range is a key limitation of this material. Herein, a novel dopant, palladium (Pd), is doped into Cs_2_AgBiBr_6_ and significantly extends the absorption to ≈1400 nm. Pd^2+^ ions are partially doped in the host lattice, most probably replacing Ag atoms and introducing a sub‐bandgap state within the host bandgap, as indicated by the combination of spectroscopical measurements and theoretical calculations. Importantly, this sub‐bandgap state extends the photoresponse of Cs_2_AgBiBr_6_ up to the NIR‐II region of 1300 nm, setting a new record for HDPs. This work demonstrates a novel and efficient dopant for HDPs and highlights the effectiveness of employing a sub‐bandgap to broaden the absorption of HDPs, shedding new light on tailoring large bandgap HDPs for NIR optoelectronic applications.

## Graphical abstract

A novel dopant of Pd is introduced into lead‐free Cs_2_AgBiBr_6_ double perovskite. Pd^2+^ enters the lattice of Cs_2_AgBiBr_6_, substitutes the crystallographic position occupied by Ag^+^, and forms a sub‐bandgap energy level in the host material. Pd‐doped Cs_2_AgBiBr_6_ shows significantly extended absorption to ≈1400 nm and NIR light response up to 1300 nm.

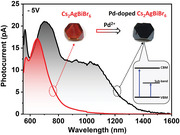

## 1 Introduction

Lead (Pb) metal halide perovskites have revolutionized the optoelectronic field over the past decade due to their superior optical and electronic properties.^[^ ^1^, ^2^ ^]^ However, state‐of‐the‐art Pb‐based perovskite optoelectronic devices suffer from toxicity and long‐term instability,^[^ ^3^, ^4^ ^]^ hindering their commercialization. Emerging lead‐free halide double perovskites (HDPs) with the formula of A_2_B^+^B^3+^X_6_ (A = Cs^+^, MA^+^; B = metal ions; X = Cl^−^, Br^−^, I^−^) are considered a promising alternative candidate due to their low toxicity, high stability, a wide range of possible combinations, and rich substitutional chemistry.^[^ ^5^, ^6^, ^7^, ^8^ ^]^ Together with their attractive optoelectronic properties, they have been successfully applied in various optoelectronic applications, including solar cells,^[^ ^9^, ^10^, ^11^ ^]^ photodetectors,^[^ ^12^, ^13^ ^]^ X‐ray detectors,^[^ ^14^, ^15^ ^]^ light‐emitting diodes^[^ ^16^ ^]^ and photocatalysis.^[^ ^17^, ^18^ ^]^ However, state‐of‐the‐art double perovskites are still limited by their large bandgaps and weak absorption.

In order to broaden the light absorption of HDPs, metal element doping/alloying is a primary approach considering the difficulty of halide mixing.^[^ ^19^, ^20^, ^21^ ^]^ This strategy mainly works by two different mechanisms, i.e., reducing the host bandgap or introducing a sub‐bandgap into the host bandgap. Generally, to substantially change the bandgap characters (such as bandgap value and direct/indirect bandgap nature) of the host material, a sufficiently high doping/alloying level is required to ensure that the dopants’ orbital states dominate the conduction band minimum (CBM) or valence band maximum (VBM). For example, the direct bandgap of 3.54 eV in Cs_2_AgInCl_6_ powder can be reduced to 2.92 eV with an indirect nature when 40% Sb^3+^ replaces In^3+^ in the lattice.^[^ ^22^ ^]^ With increasing Sb composition, the Sb 5s^2^ state makes an increasing contribution to the valence bands; in addition, Sb 5p_1/2_ also gradually replaces the In 5s^0^ state as the CBM. These two effects collectively decrease the bandgap of Cs_2_AgInCl_6_ by Sb^3+^ doping. Similarly, by incorporating ≈50% Bi^3+^ in Cs_2_AgInCl_6_, the direct bandgap of 3.67 eV was changed to an indirect bandgap of 2.82 eV.^[^ ^23^ ^]^ We have previously reported a more efficient dopant Fe^3+^, which could turn the optical bandgap of Cs_2_AgInCl_6_ crystal from 2.8 to 1.6 eV through 0 to 100% Fe^3+^ alloying.^[^ ^24^ ^]^ However, a high level of metal doping/alloying, which is required to tune the bandgap, is highly determined by the structural stability of the resulting alloying/doping system. These successful cases (as discussed above) are very limited; in most cases, the alloying/doping can only reach a low level (below 5%), limiting the practicality of this approach.

Compared to reducing the host bandgap, introducing a sub‐bandgap into the host bandgap offers a simple yet efficient approach, requiring a lower doping level while effectively expanding the absorption range.^[^ ^25^, ^26^, ^27^, ^28^ ^]^ Taking the benchmark HDP Cs_2_AgBiBr_6_ as an example, we noticed that only 1% Cu^+^/Cu^2+^ can be doped in the host lattice. Interestingly, this small doping level can expand the absorption edge from ≈610 to 860 nm, arising from the formation of sub‐bandgap rather than bandgap narrowing.^[^ ^28^ ^]^ Our first‐principles density functional theory (DFT) calculations further revealed that the sub‐bandgap in Cu‐doped Cs_2_AgBiBr_6_ was ascribed to the doping‐induced defect states rather than a direct effect of Cu ions on the electronic structure. However, this type of sub‐bandgap formation by uncertain defect states is difficult to control and the absorption extension is limited. Therefore, exploring other dopants with orbitals that can directly contribute to the sub‐bandgap state is highly desirable, which could enhance and engineer the sub‐bandgap state in the host materials, further expanding the absorption range.

Herein, we employ a novel dopant Pd^2+^ to alloy the benchmark HDP Cs_2_AgBiBr_6_. The incorporation of ≈5.5% Pd^2+^ into the lattice significantly broadens the absorption edge from 570 nm to the near‐infrared II (NIR‐II) region of ≈1400 nm, which is so far the longest absorption range for both lead‐based and lead‐free perovskites. Our theoretical calculations reveal that the broadened absorption originated from the formation of a sub‐bandgap state which is dominated by the Pd (d) orbitals inside the pristine bandgap. The Pd‐doped Cs_2_AgBiBr_6_ photodetector exhibits a noticeable photoresponse under 1300 nm excitation, indicating its potential application in intermediated band solar cells, NIR photodetectors, and bioimaging.

## 2 Results and Discussion

**Figure 1:**
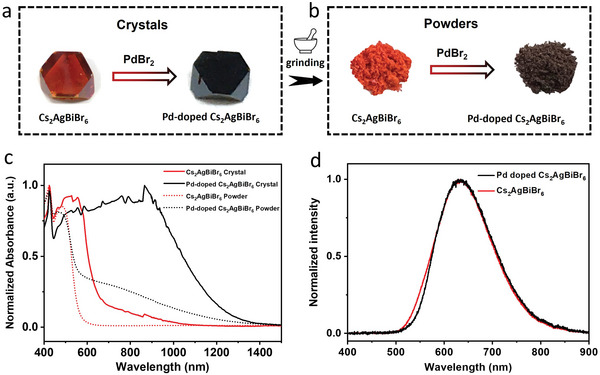
Optical images of a) pristine Cs_2_AgBiBr_6_ and Pd‐doped Cs_2_AgBiBr_6_ crystals and b) the fine powders obtained from grinding the crystals. c) Normalized UV–vis–NIR absorption of pristine Cs_2_AgBiBr_6_ and Pd‐doped Cs_2_AgBiBr_6_ crystals and powders. The detector was switched at 860 nm. d) Normalized photoluminescence of pristine Cs_2_AgBiBr_6_ and Pd‐doped Cs_2_AgBiBr_6_ crystals.

The high‐quality and large‐size (5–10 mm) Pd‐doped Cs_2_AgBiBr_6_ single crystals are synthesized using a modified hydrothermal method with CsBr, AgBr, BiBr_3,_ and PdBr_2_ in HBr acid (more details in the Experimental Section of the Supporting Information). As the molar ratio of Pd ions within the final crystals could significantly differ from its precursor solutions, we first perform the inductively coupled plasma optical emission spectroscopy (ICP‐OES) measurement to determine the molar ratio values. The results reveal that a maximum of 5.5% Pd can be incorporated into the final crystals (Table S1, Supporting Information). Unless otherwise specified, all the target samples are based on 5.5% Pd‐doped Cs_2_AgBiBr_6_ for the following experiments. Notably, this small amount of Pd doping can dramatically alter the crystal color from red to black, suggesting an expanded absorption range in Pd‐doped Cs_2_AgBiBr_6_ (**Figure** **1**a). We grind the large crystals into fine powders and find a similar color change behavior from orange to black (Figure 1b).

We measure the UV–vis–NIR absorbance to qualify the color difference between the undoped and Pd‐doped Cs_2_AgBiBr_6_ in both crystal and powder samples (Figure 1c). The powder samples of undoped and Pd‐doped Cs_2_AgBiBr_6_ both show a sharp absorption edge at ≈570 nm, implying that the Pd doping does not change the bandgap. The difference is that upon the Pd doping, a strong tail absorption is observed, extending to ≈1400 nm. To the best of our knowledge, this is the widest reported absorption range for any known metal halide perovskites to date (Table S2, Supporting Information). Similar absorption extension is also clear in the crystal samples, where the absorption edges of both samples show a redshift compared with the powder samples. The redshift can be attributed to the low absorption coefficient induced by the indirect bandgap, resulting in a significant thickness dependency on the absorbance. Figure S1 (Supporting Information) shows the absorption spectra of Pd‐doped Cs_2_AgBiBr_6_ at other doping concentrations, which all have similar broadened absorption edges, but the tail absorption is enhanced with increasing doping concentration. The photoluminescence (PL) measurements (Figure 1d) of both undoped and Pd‐doped Cs_2_AgBiBr_6_ demonstrate a similarly broad PL peak centered at around 634 nm, indicating a similar bandgap of ≈1.95 eV. We further perform the time‐correlated single‐photon counting (TCSPC) measurement for the two samples, and Pd‐doped Cs_2_AgBiBr_6_ exhibits a shorter PL lifetime than the undoped one, implying more defects inside (Figure S2, Supporting Information).

**Figure 2:**
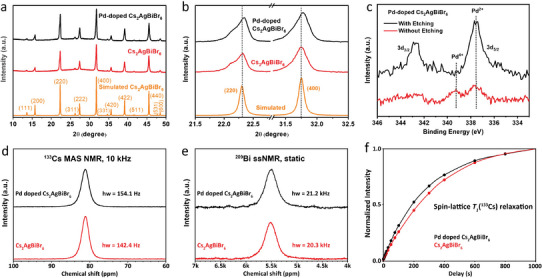
a) Powder XRD patterns of Cs_2_AgBiBr_6_ and Pd‐doped Cs_2_AgBiBr_6_. b) The enlarged view of the (220) and (400) diffraction peaks. c) XPS Pd 3d spectrum of Pd‐doped Cs_2_AgBiBr_6_ with/without etching. Experimental ^133^Cs MAS NMR spectra d) recorded at 10 kHz spinning speed and ^209^Bi ssNMR spectra e) acquired at static conditions of pristine Cs_2_AgBiBr_6_ and Pd‐doped Cs_2_AgBiBr_6_. f) Experimental ^133^Cs *T*_1_ saturation recovery build‐up curves of pristine and Pd‐doped Cs_2_AgBiBr_6_ perovskites.

To understand the Pd doping behavior, we investigate the structure evolution by performing a series of structural and spectroscopic measurements. Single‐crystal X‐ray diffraction (SCXRD) data reveal that both samples crystallize in the cubic space with the $Fm\bar{3}m$ space group (Table S3, Supporting Information), indicating that the dopant does not change the host crystal structure. Furthermore, we observe the lattice shrink after Pd doping with the lattice parameter decreasing from 11.2288 to 11.1984 Å. This fact can be understood by the smaller ionic radii of Pd (Pd^2+^(0.86 Å)/ Pd^4+^(0.62 Å)) compared with Ag^+^ (1.15 Å) and Bi^3+^ (1.03 Å).^[^ ^29^ ^]^ These results are further confirmed by the powder X‐ray diffraction (PXRD) data in **Figure** **2**a,b. Both undoped and Pd‐doped samples show nearly identical features to the simulated one without any impurity peaks, and all diffraction peaks including the dominant diffraction peaks (220) and (400) shift toward the higher angle side after Pd doping. Notably, the ionic radii of Pd are comparable to those of Ag^+^ and Bi^3+^, and its ability to form stable chemical bonds with Br^−^ are the two critical factors for successful doping.

Considering that Pd ions mainly have two stable chemical states of 2+ and 4+, the X‐ray photoelectron spectroscopy (XPS) measurement is employed to investigate the valence state of Pd in the lattice. Without etching, two weak Pd 3d_5/2_ signals with binding energies of 337.6 and 339.25 eV are detected in Pd‐doped Cs_2_AgBiBr_6_, which are attributed to Pd^2+^ and Pd^4+^, respectively (Figure 2c).^[^ ^30^ ^]^ It is worth noting that only a Pd^2+^ signal is present after etching, suggesting that Pd^4+^ primarily originates from the surface oxidation of Pd^2+^ (Figure 2c and the full spectrum in Figure S3, Supporting Information). We then conclude that Pd^2+^ is doped in the host lattice. Note that the valence state of doped Pd is different from Ag^+^ and Bi^3+^, this could be one of the reasons why a limited amount of Pd^2+^ (≈5.5% at maximum) can be successfully doped in the final crystal lattices.

The remaining question is the Pd doping position in the host lattice. Solid‐state nuclear magnetic resonance (ssNMR) is a powerful tool to determine the atomic‐level structure of solid materials. We perform ^133^Cs and ^209^Bi ssNMR analysis on pristine and Pd‐doped Cs_2_AgBiBr_6_. As shown in Figure 2d, only one symmetric peak at δ_iso_ = 81.2 ± 0.5 ppm in the ^133^Cs magic angle spinning (MAS) NMR spectra is observed, confirming one crystallographic position of the Cs^+^ ions in both systems. This is because both samples have the same cubic crystalline structure as determined by SCXRD. Notably, the ^133^Cs peak shows a noticeable broadening of the half‐width from 142.4 to 154.1 Hz after Pd doping, implying that Pd is doped into the lattice and affects the nearest Cs^+^ nuclei. Usually, the dopant in the lattice would introduce new signals owing to the influenced chemical environment of the detected nuclei. Here, the absence of new, specific signal(s) in ssNMR spectra of the doped system could be explained by the “heavy‐atom effects on the shielding of the heavy atom itself” (HAHA effect) phenomenon.^[^ ^31^ ^]^ This implies that one heavy atom has replaced another, and the local effect of one heavy atom on another (HAHA effect) makes any change in NMR chemical shift values practically cancel out.^[^ ^32^ ^]^ Additional structural information is provided from ^209^Bi ssNMR spectra, where a very broad (few hundred ppm) signal at ca. δ_iso_ = 5500 ppm is detected in both cases (Figure 2e). The half‐width broadening of ^209^Bi ssNMR signal in Pd‐doped Cs_2_AgBiBr_6_ further confirms the existence of Pd dopant in the structure. Considering that the distance between two Bi atoms (4‐bonds) is twice as long as the Bi‐Ag distance, the effect on the ^209^Bi ssNMR spectra at replacing Bi atoms would be minimal, which implies that Pd replaces Ag in the structure.

To further investigate the possible distribution of doped Pd atoms, we move forward to determine the ^133^Cs spin‐lattice relaxation (*T*_1_) times, as small changes in the investigated nuclei's local environment will significantly impact the *T*_1_ relaxation times.^[^ ^24^ ^]^ The obtained ^133^Cs spin‐lattice relaxation decays show a slightly accelerated ^133^Cs spin‐lattice relaxation time from 376 to 281 s in the Pd‐doped Cs_2_AgBiBr_6_ system, as shown in Figure 2f and Table S4 (Supporting Information). Although the single‐exponential analysis of the recorded ^133^Cs spin‐lattice relaxation decays could be sufficient, demonstrated by the relatively low values of statistical parameters standard deviations (*SD*) and residual sum of squares (*RSS*) (Table S4, Supporting Information), the physically more realistic model involving application of double‐exponential functions is used for the Pd‐doped system. We additionally apply a set of double‐exponential models differing in initial estimates of *T*_1_ relaxation times. Averaged values of the obtained best fits are summarized in Table S5 (Supporting Information). The double‐exponential model reveals the existence of second‐phase Cs atoms in the doped system, which exhibits a shorter *T*_1_(^133^Cs) _II_ relaxation time of 180 s. Compared with *T*_1_(^133^Cs)_I_ of 388 s, the second component *T*_1_(^133^Cs)_II_ of 180 s is not significantly shortened, implying that the interactions with Cs atoms are averaged and spread over a larger space. Combined with the rapidly relaxing fraction of Cs atoms (second component) of up to 13–31%, a uniform distribution/dispersion of Pd^2+^ in the host lattice is supposed.

**Figure 3:**
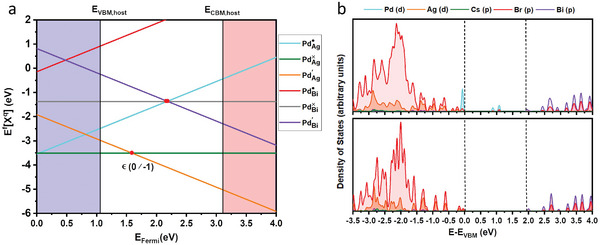
a) Defect transition level diagram and b) Density of states for the pristine (bottom) and (−1) charged 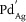 defect structures (top), respectively.

To understand the role of the dopant in the formation of sub‐bandgap states and its influence on the electronic structure of the material, we perform electronic structure calculations at the level of density functional theory (DFT). For a more accurate description of the electronic structure, we employ the HSE06 hybrid function including spin‐orbit coupling effects, which matches the experimentally observed bandgap within 0.1 eV. By modeling possible substitutional defects in a Special Quasi‐random Structure (SQS),^[^ ^33^ ^]^ we screened several possible neutral and charged defect states when the Pd dopant substitutes the B^+^ = Ag sublattice, B^3+^ = Bi sublattice or both Ag and Bi sublattices simultaneously, with the concentration of Pd^2+^ approximating the experimentally determined value (See Table S1, Supporting Information). The transition level diagram obtained illustrating the substitutional defect landscape is shown in **Figure** **3**a. The stable Pd‐substitutional defects with Fermi levels ranging across the host‐bandgap are found to be neutral spin‐polarized defect ${\mathrm{Pd}}_{\mathrm{Ag}}^{\times }$ and negatively charged (−1) defect, 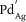. All other defects are energetically >1 eV higher and can hardly provide a valuable contribution. The defects where Pd^2+^ substitutes both Ag and Bi sublattices are found to be relatively unstable in this case. The corresponding density of states for the defect structure we predict to be predominant, as well as the host material, are illustrated in Figure 3b. It is clear that the mid‐gap states are dominated by the dopant Pd(d) orbitals which lie ≈0.9 eV above the main valence band with respect to the host material's bandgap. It is also worthwhile to note that these mid‐gap Pd(d) states also hybridize with the Br(p) states as apparent from the density of states. From the transition level diagram and the corresponding density of states, we can conclude that Pd‐doping at this concentration results in a negatively charged (−1) substitutional defect 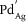 where Pd^2+^ occupies lattice sites in the Ag sublattice and contributes to the formation of sub‐bandgap states. Furthermore, within the explored range of Pd‐dopant concentration, we expect this defect type to be dominant, and the position of the sub‐bandgap state relative to band edges to be minimally affected by the dopant concentration. At the same time, a higher content of Pd^2+^ dopants in the host structure could increase the density of Pd(d) orbitals in the host's band structure. This increased Pd(d) orbital density can facilitate a larger number of band‐to‐band transitions, which is expected to result in enhanced tail absorption intensity. These insights could effectively explain the trend of the doping concentration‐dependent absorption spectra in Figure S1 (Supporting Information).

**Figure 4:**
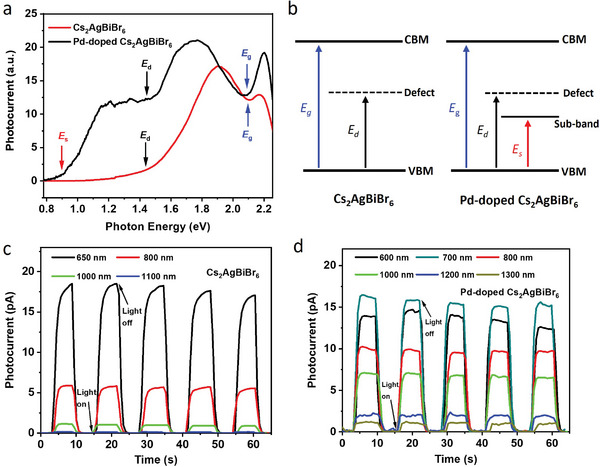
The photocurrent of the pristine Cs_2_AgBiBr_6_ and Pd‐doped Cs_2_AgBiBr_6_ single‐crystal photodetectors at a 5 V bias. b) Schematic illustration of photo‐excitation processes in pristine Cs_2_AgBiBr_6_ and Pd‐doped Cs_2_AgBiBr_6_. The light on/off switching performance of the pristine Cs_2_AgBiBr_6_ c) and Pd‐doped Cs_2_AgBiBr_6_ d) single‐crystal photodetectors at a 5 V bias under various excitation wavelengths.

Now, the Pd doping behavior and sub‐bandgap formation mechanism are well understood; however, it remains unclear whether these absorptions can generate photocarriers, which is a key requirement for potential optoelectronic applications such as solar cells, photodetectors, or bioimaging. We therefore fabricate simple single‐crystal photodetectors with a photoconductive planar structure by depositing two gold electrodes onto the surfaces of pristine and Pd‐doped Cs_2_AgBiBr_6_ crystals (Figure S4, Supporting Information). Monochromatic light with wavelengths ranging from 400 to 1600 nm is used to excite both devices at a 5 V bias. **Figure** **4**a compares the photocurrent of both devices as a function of excitation energy, with the wavelength‐dependent responsivity shown in Figure S5 (Supporting Information). While the photon flux is different at different energies in Figure 4a, this figure provides clear information for us to understand different transitions. In the undoped Cs_2_AgBiBr_6_ device, we notice two rises of photocurrent at ≈1.45 and 2.1 eV, respectively. The energy of the first rise is much lower than its bandgap energy of ≈1.95 eV, suggesting the presence of a defect state in the bandgap, where photocarriers can be generated through a transition between the defect state and either the valence or conduction band. This is illustrated in Figure 4b, where a transition between a defect level and the valence band is shown as an example. This relatively deep defect state in the undoped Cs_2_AgBiBr_6_ possibly arises from crystal surface defects, Ag/Bi disorder, or lattice vacancies (such as Bi vacancies).^[^ ^34^, ^35^ ^]^ The second photocurrent increase at ≈2.1 eV, slightly above the bandgap energy, can be attributed to photogenerated carriers primarily originating from band‐to‐band transitions that require above‐bandgap energy excitation, consistent with our previous report.^[^ ^28^ ^]^ We note that the below‐bandgap peak intensity is higher than the above‐bandgap one (the same can be observed for the Pd‐doped sample), possibly due to a combined effect of two reasons: 1) the photon flux of our light source increases with decreasing energy (down to 0.92 eV); 2) low‐energy light can penetrate deeper into the sample, helping to generate more charge carriers and minimizing the effect from surface recombination.^[^ ^36^ ^]^

The Pd‐doped device also exhibited a similar increase in photocurrent at ≈2.1 eV due to band‐to‐band carrier generation, confirming the fact that the bandgap remains relatively unchanged after Pd doping. Meanwhile, a photocurrent rise at ≈1.45 eV is also detected, suggesting the presence of a similar defect state in the Pd‐doped Cs_2_AgBiBr_6_ crystal as in the undoped one. Notably, an additional and relatively strong photoresponse at ≈0.9 eV is found in the Pd‐doped Cs_2_AgBiBr_6_ device, while it is absent in the undoped device (Figure 4a). This result provides solid evidence of photo‐carrier generation between the sub‐bandgap and the valence or conduction band, as illustrated in Figure 4b.

Inspired by the noticeable defect state and sub‐bandgap‐induced photocarriers, it is feasible to use the undoped and Pd‐doped Cs_2_AgBiBr_6_ with large bandgaps to detect NIR photons. Figure 4c shows the light‐switching characteristics of the undoped Cs_2_AgBiBr_6_ crystal device at a 5 V bias under various excitation wavelengths, extending to 1100 nm. The photocurrent response becomes negligible when the excitation wavelength reaches 1100 nm, due to its low photon energy falling well below the bandgap and defect state energy. For the Pd‐doped Cs_2_AgBiBr_6_, the light detection can be extended to the NIR‐II light of 1300 nm excitation, where a distinct photocurrent response can still be detected (Figure 4d). This result represents the longest photoresponse for perovskites reported so far (Table S6, Supporting Information) and confirms the potential of employing the sub‐bandgap for optoelectronic applications. Although further optimization is still required to improve carrier mobility, passivate the surface defects, minimize contact resistance, etc., before achieving competitive device performance compared to existing state‐of‐the‐art NIR light‐detecting materials, these findings demonstrate an excellent example of the doping effect in a double perovskite with a large bandgap and how it can be tailored for new optoelectronic applications. We also note that during the preparation of the manuscript, a similar approach of using Fe^3+^/Ru^3+^ dopants was reported, extending the absorption edge and photoresponse of Cs_2_AgBiBr_6_ to the NIR range (980 nm),^[^ ^37^, ^38^ ^]^ which also supports the effectiveness of the strategy of introducing a dopant‐dominated sub‐bandgap.

Stability is an important parameter to evaluate the practical use of HDPs for optoelectronic applications. We have therefore investigated the environmental, thermal, and photodetector device stability of undoped and Pd‐doped Cs_2_AgBiBr_6_ by time‐dependent PXRD, thermogravimetric analysis, and time‐dependent photocurrent measurements. As shown in Figure S6 (Supporting Information), no noticeable decomposition signals appear after 200 days of storage in the air for both samples, indicating their excellent environmental stability. Thermogravimetric analysis suggests that Pd‐doped Cs_2_AgBiBr_6_ has better thermal stability with a slightly increased decomposition temperature from 433 to 448 °C (Figure S7, Supporting Information). Moreover, differential scanning calorimeter (DSC) measurements indicate that both materials have no phase transitions at the temperature range from 32 to 400 °C (Figure S8, Supporting Information). Regarding the photodetector device stability, we perform the time‐dependent photocurrent measurements and observe no photocurrent changes after two weeks of storage in ambient conditions for both pristine and Pd‐doped Cs_2_AgBiBr_6_ devices (Figure S9, Supporting Information). The excellent environment, thermal, and device stability make the Pd‐doped Cs_2_AgBiBr_6_ a promising candidate for potential long‐period optoelectronic applications such as intermediated band solar cells, NIR photodetectors, and bioimaging.

## 3 Conclusion

In summary, we have explored a novel dopant Pd to dope the benchmark HDP Cs_2_AgBiBr_6_ and successfully extended its absorption from 570 to ≈1400 nm. A range of spectroscopical measurements indicate that Pd^2+^ ions are homogeneously distributed in the host structure, partially replacing the Ag sublattice and shrinking the crystal structure. Furthermore, the Pd(d) orbital states contribute to the formation of a sub‐bandgap within the host bandgap, as revealed by DFT calculations, resulting in significantly extended absorption in Pd‐doped Cs_2_AgBiBr_6_. Importantly, we observe photo‐carrier generation as a result of the transition between the sub‐bandgap and the valence or conduction band, extending the photoresponse region to 1300 nm, which is the longest photoresponse region reported for both lead‐based and lead‐free perovskites to date. In addition, Pd‐doped Cs_2_AgBiBr_6_ exhibits excellent environment stability and enhanced thermal stability. Our findings pave the way for engineering the sub‐bandgap state in double perovskites, unlocking their potential for NIR and other high‐efficiency optoelectronic applications.

## Conflict of Interest

The authors declare no conflict of interest.

## Supporting information



Supporting Information

## Data Availability

The data that support the findings of this study are available from the corresponding author upon reasonable request.
