# Supplementary material for: Palladium‐Doped Cs2AgBiBr6 with 1300 nm Near‐Infrared Photoresponse
Source: Small. 2024 Sep 20;20(49):2404188. doi: 10.1002/smll.202404188 (PMC11618710; doi:10.1002/smll.202404188)
Supplement: Supplementary file 1 — Supporting Information [file SMLL-20-2404188-s001.pdf]

NANO · MICRO
small

Supporting Information

for *Small*, DOI 10.1002/smll.202404188

Palladium-Doped $Cs_2AgBiBr_6$ with 1300 nm Near-Infrared Photoresponse

Hongwei Lei, Utkarsh Singh, Fuxiang Ji, Tinghao Lin, Libor Kobera, Yuequn Shang, Xinyi Cai, Weihua Ning, Andrii Mahun, Sabina Abbrent, Zuojun Tan*, Jiri Brus, Dehui Li, Sergei I. Simak, Igor A. Abrikosov and Feng Gao**

Supporting information

Palladium-doped $Cs_2AgBiBr_6$ with 1300 nm near-infrared photoresponse

Hongwei Lei, Utkarsh Singh, Fuxiang Ji, Tinghao Lin, Libor Kobera, Yuequn Shang, Xinyi Cai, Weihua Ning, Andrii Mahun, Sabina Abbrent, Zuojun Tan,* Jiri Brus, Dehui Li, Sergei I. Simak, Igor A. Abrikosov, and Feng Gao**

Dr. H. W. Lei, Prof. Z. J. Tan
College of Engineering
Huazhong Agricultural University
Wuhan, 430070, China
Email: tzj@mail.hzau.edu.cn

Dr. H. W. Lei, U. Singh, Dr. F. X. Ji, Dr. Y. Q. Shang, Dr. X. Y. Cai, Dr. W. H. Ning, Prof. S. I. Simak, Prof. I. A. Abrikosov, Prof. F. Gao
Department of Physics, Chemistry and Biology (IFM)
Linköping University
Linköping, SE-581 83, Sweden
Email: fuxi@zhaw.ch, feng.gao@liu.se

Prof. Sergei I. Simak
Department of Physics and Astronomy
Uppsala University
Uppsala, SE-751 20, Sweden

T. Lin, Prof. D. H. Li
School of Optical and Electronic Information and Wuhan National Laboratory for Optoelectronics
Huazhong University of Science and Technology,
Wuhan, 430074, China

Dr. L. Kobera, A. Mahun, Dr. S. Abbrent, Dr. J. Brus
Institute of Macromolecular Chemistry of the Czech Academy of Sciences
Heyrovskeho nam. 2, Prague 162 00, Czech Republic

1. Experimental Section

Materials Synthesis and Films Fabrication

All the chemicals used were purchased from Sigma-Aldrich without any further purification. For pristine $Cs_2AgBiBr_6$ crystals, solid CsBr (213 mg, 1 mmol), $BiBr_3$ (224 mg, 0.5 mmol) and AgBr (94 mg, 0.5 mmol) were mixed in 6 mL of 48% HBr and then transferred into a 25 cm^3 Teflon-lined autoclave. The autoclave was sealed and placed in the oven where it was heated to 120 °C for 24 h. After being slowly cooled to room temperature at a rate of 1 °C h^{-1}, red octahedral single crystals were achieved. For Pd-doped $Cs_2AgBiBr_6$ crystals, $PdBr_2$ powders with different mol ratios (0.016%-60%) to $BiBr_3$ were added to the precursor solution, respectively. Further increasing the $PdBr_2$ ratio in precursor solution (> 60%) will not generate any crystals. To obtain large-size (5-10 mm) Pd-doped $Cs_2AgBiBr_6$ single crystals, $PdBr_2$ powders were first dissolved in 48% HBr to prepare a solution with a concentration of 5 mg/mL, which was then added to the precursor solution. Other synthesis parameters were the same as that applied for pristine $Cs_2AgBiBr_6$. The yield is ≈ 75%.

Physical measurements

The XRD patterns of the powders and thin films were recorded with an X'Pert PRO X-ray diffractometer using Cu Kα1 irradiation (λ = 1.5406 Å). The UV-Vis reflectance or absorption spectra were measured with Shimadzu UV-3600. Steady-state photoluminescence spectra were recorded with a 405 nm laser and an Andor spectrometer (Shamrock sr-303i-B, coupled to a Newton EMCCD detector). Time-correlated single photon counting (TCSPC) measurements were carried out by using an Edinburgh Instruments FL1000 with a 405 nm pulsed picosecond laser (EPL-405). X-ray photoelectron spectroscopy (XPS) spectra were acquired using a photoelectron spectroscopy model Thermo Scientific, ESCLAB 250Xi. Samples were sputter-cleaned in the XPS chamber by lower energy Ar^+ for 30s to remove the atmospheric contamination before testing and the vacuum pressure was kept lower than 10^{-7} Pa in the

analysis chamber. Inductively coupled plasma optical emission spectroscopy (ICP-OES) was performed using the Thermo Scientific iCAP 7400. The samples were digested entirely in diluted aqua regia solution using by a microwave digestion system (Multiwave 7000) to detect the concentrations of Pd. The same batch of samples was additionally digested in HNO_3 or in ammonium hydroxide to further measure the concentration of Bi, Cs, and Ag, respectively. Thermogravimetric (TG) and differential scanning calorimetry (DSC) experiments were performed with a DSC 300 analyzer (NETZSCH, Selb, Germany) at a warming rate of 10 K min^{-1} under a nitrogen atmosphere.

Optical absorption determination of Crystals

The reflectance spectra of crystal samples we obtained (Figure S10) were converted to pseudo-absorbance spectra using the Kubelka-Munk transform.[1]

$\alpha \approx (1\text{-}R)^2/2R$

where α = pseudo-absorbance and R = reflectance.

Solid-state nuclear magnetic resonance (ssNMR)

The ssNMR spectra were recorded at 11.7 T using a Bruker AVANCE III HD spectrometer. The 4 mm cross-polarization magic angle spinning (CP/MAS) probe was used for ^{133}Cs and ^{209}Bi experiments at Larmor frequency of $\nu(^{133}\text{Cs})$ = 65.611 MHz and $\nu(^{209}\text{Bi})$ = 80.858 MHz, respectively. ^{133}Cs MAS NMR experiments were collected at 10 kHz spinning speed without ^{1}H decoupling. The recycle delay was 600 s for all ssNMR experiments. The ^{133}Cs chemical shift was calibrated using solid CsCl (^{133}Cs: 228.1 ppm). The pulse length was set to 2.2 μs at 100 W for maximal signal intensity. The ^{133}Cs spin-lattice relaxation (T_1) decays were measured using the standard saturation recovery pulse system consisting of a train of 200 short ($^{\pi}/_{2}$) pulses each separated by 50 ms delay. The delay times for ^{133}Cs spin-lattice relaxation consisting of 18 increments varied between 0.1 and 1000 s with 4 scans used for each increment. The length of $^{\pi}/_{2}$ pulse was 2.4 μs. No ^{1}H decoupling was applied during the data acquisition. The obtained relaxation build-up curves were fitted by single- and double-

exponential functions (see Tables S4 and S5, respectively). The double-exponential model was used for the analysis of the relaxation behavior of Pd-doped $Cs_2AgBiBr_6$ system which resulted in a clear decrease in statistical parameters (standard deviations (SD) and residual sum of squares (RSS)). The ^{209}Bi chemical shift was calibrated using a saturated solution of $Bi(NO_3)_3·5H_2O$ in concentrated HNO_3 (^{209}Bi: 0.0 ppm).[2] The pulse length was 9.0 μs at 100 W at maximal signal intensity. ^{209}Bi NMR experiments were collected at static conditions using single-pulse NMR experiments. To compensate for the frictional heating of the spinning samples, all NMR experiments were measured under active cooling. The sample temperature was maintained at 298 K and the temperature calibration was performed on $Pb(NO_3)_2$ using a calibration procedure described in the literature.[3] The dried sample was packed into ZrO_2 rotors and subsequently stored at room temperature. All NMR spectra were processed using the Top Spin 3.5 pl2 software package.

Photoconductivity measurement

The pristine $Cs_2AgBiBr_6$ and Pd-doped $Cs_2AgBiBr_6$ single-crystal devices were made by evaporating two gold electrodes onto their top (111) surfaces. The device area of both photodetectors is 0.02 cm^2. Photoconductivity measurements were measured by a home-built photoconductivity measurement system. Here, a halogen lamp was used as the light source which is dispersed by a monochromator (Horiba JY iHR320). The monochromatic output light was then collimated by two lens and finally shined onto the device. The photocurrent was collected by a low-noise amplifier (Stanford SR570) and a lock-in amplifier (Stanford SR830).

Theoretical Calculations

Electronic structure calculations: All density functional theory (DFT) calculations were performed in the framework of the projector augmented wave (PAW)[4] method, as implemented in the Vienna ab initio simulation package (VASP).[5–7] A cutoff energy of 450 eV was used for the plane wave expansion of the Kohn-Sham

orbitals. The exchange-correlation energy was approximated using the PBEsol [8] and hybrid HSE06 [9] functionals, including spin orbit coupling (SOC). The defect structures were modelled using a Special Quasi-Random Structure (SQS) [10] approach, with 160 atom supercells approximating a concentration of 6.25 at. % of Pd, which is close to the experimentally determined value of 5.5 at. %. The lattice constant was fixed at the relaxed PBEsol equilibrium value (11.17 Å) and internal coordinates were relaxed with the HSE06 functional with a k-point density of 0.30 $Å^{-1}$ and a Gaussian smearing with a width of 0.05 eV until the residual forces < 10^{-2} eV $Å^{-1}$ were achieved.

Defect formation energy: The defect formation energy, $E^f[X^q]$, corresponding to a defect of type X having charge state q was calculated using the following expression

$$E^f[X^q] = E_{tot}[X^q] - E_{tot}[Cs_2AgBiBr_6, bulk] - \sum_i n_i\mu_i + qE_F + E_{corr.}$$

Here, $E_{tot}[X^q]$ and $E_{tot}[Cs_2AgBiBr_6, bulk]$ are the HSE06 total energies for the defect supercell and pristine bulk supercell respectively. Positive n_i represents the number of atoms added and negative n_i represents the number of atoms removed from the pristine structure. $E_{corr.}$ includes finite-size corrections, band filling corrections and band edge shifting corrections for a particular defect X^q. The range of chemical potential values where the Pd-doping is possible were calculated using the CPLAP [11] package by considering 48 competing phases for the Cs-Ag-Bi-Br-Pd convex hull, including the elemental phases. The calculations for total energy these competing phases were carried out at the same level of theory and with the same parameters as the defect supercells. The Freysoldt-Neugebauer-Van de Walle (FNV) correction scheme [12] scheme was used to correct for finite-size corrections. The transition level diagram was then constructed by calculating $E_{tot}[X^q]$ for each defect type considered in the study and plotted as a function of E_F, which is the Fermi level of the defect system referenced to the valence band maxima of the host structure.

2. Supporting Figures and Tables

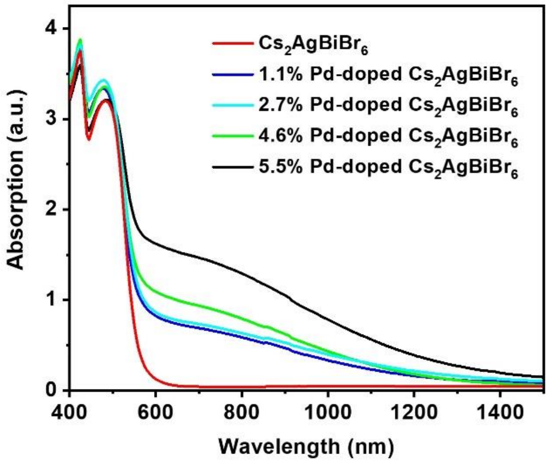

Figure S1. UV-vis-NIR absorption of pristine $Cs_2AgBiBr_6$ and various Pd-doped $Cs_2AgBiBr_6$ double perovskites.

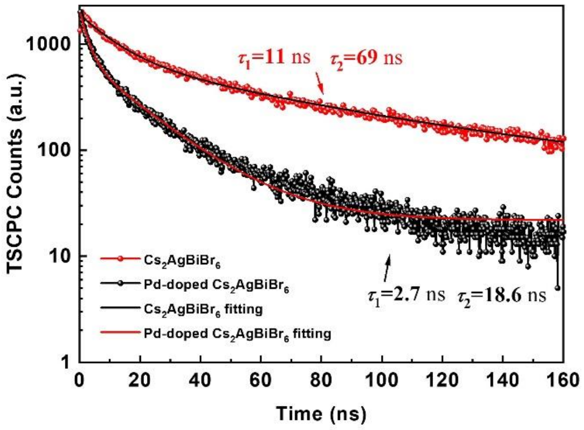

Figure S2. Time-resolved PL of pristine $Cs_2AgBiBr_6$ and Pd-doped $Cs_2AgBiBr_6$.

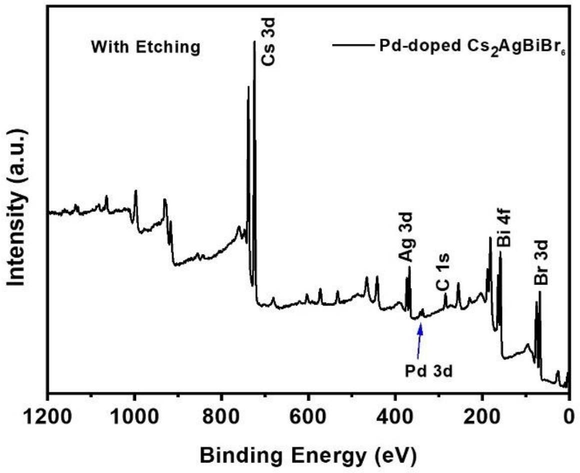

Figure S3. Full-range XPS spectrum of Pd-doped $Cs_2AgBiBr_6$ with etching.

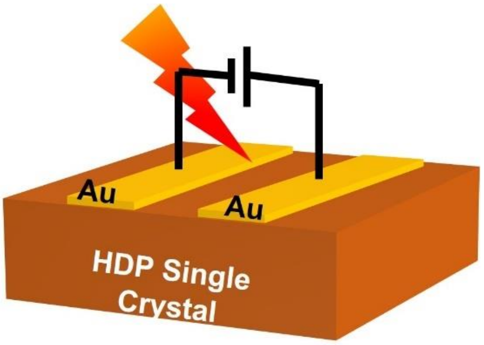

Figure S4. schematic diagram of the undoped $Cs_2AgBiBr_6$ and Pd-doped $Cs_2AgBiBr_6$ single-crystal photodetectors.

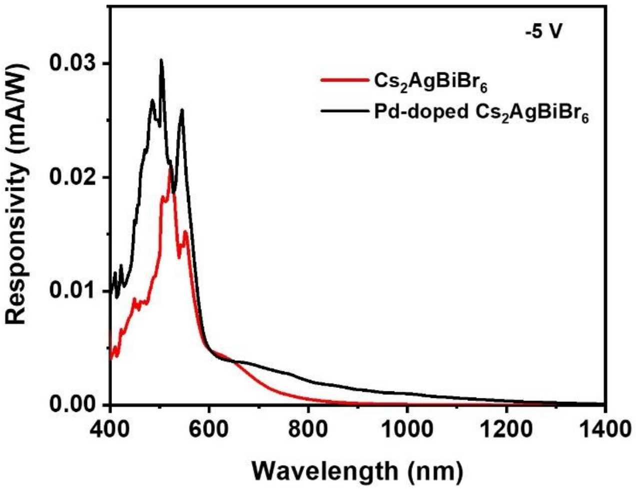

Figure S5. Wavelength-dependent responsivity of the pristine $Cs_2AgBiBr_6$ and Pd-doped $Cs_2AgBiBr_6$ single-crystal photodetectors under 5 V bias.

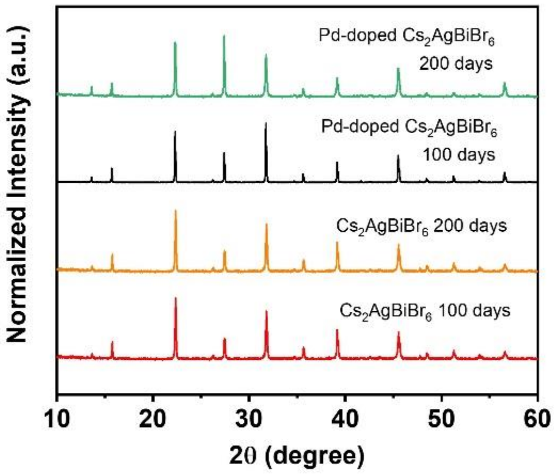

Figure S6. Time-dependent PXRD of pristine and Pd-doped $Cs_2AgBiBr_6$ after exposure to ambient conditions.

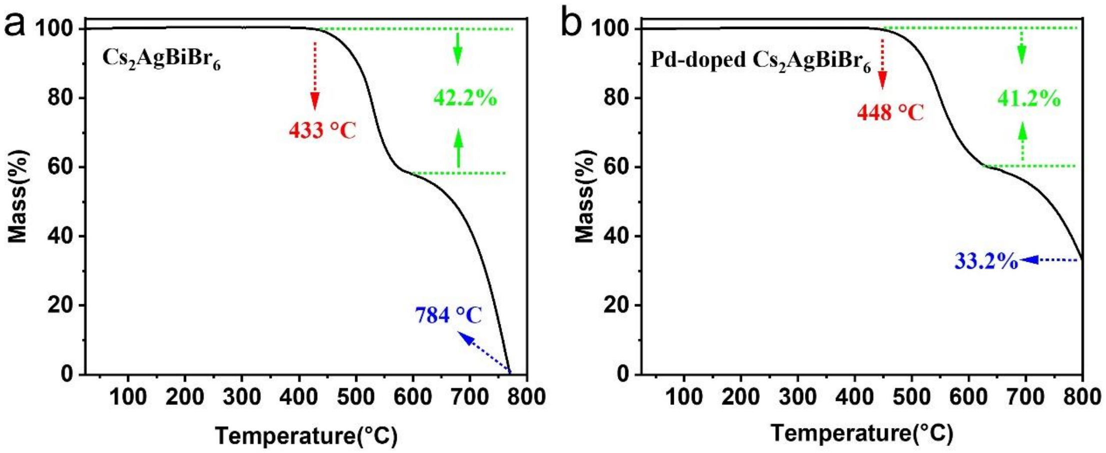

Figure S7. Thermogravimetric curves of pristine a) and Pd-doped b) $Cs_2AgBiBr_6$ powder.

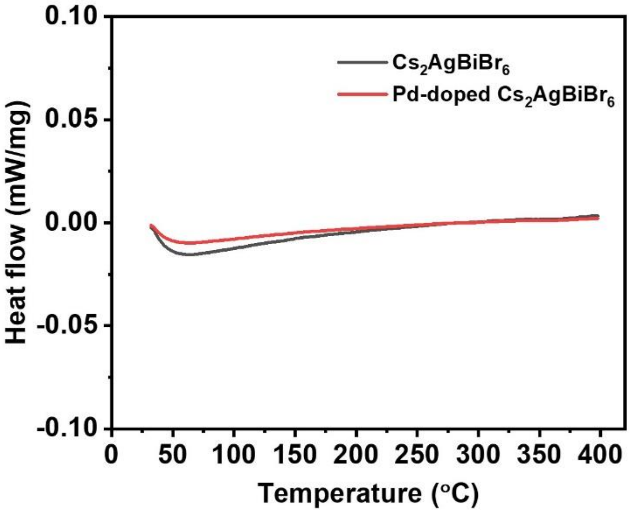

Figure S8. Differential scanning calorimeter (DSC) curves of pristine and Pd-doped $Cs_2AgBiBr_6$ powder.

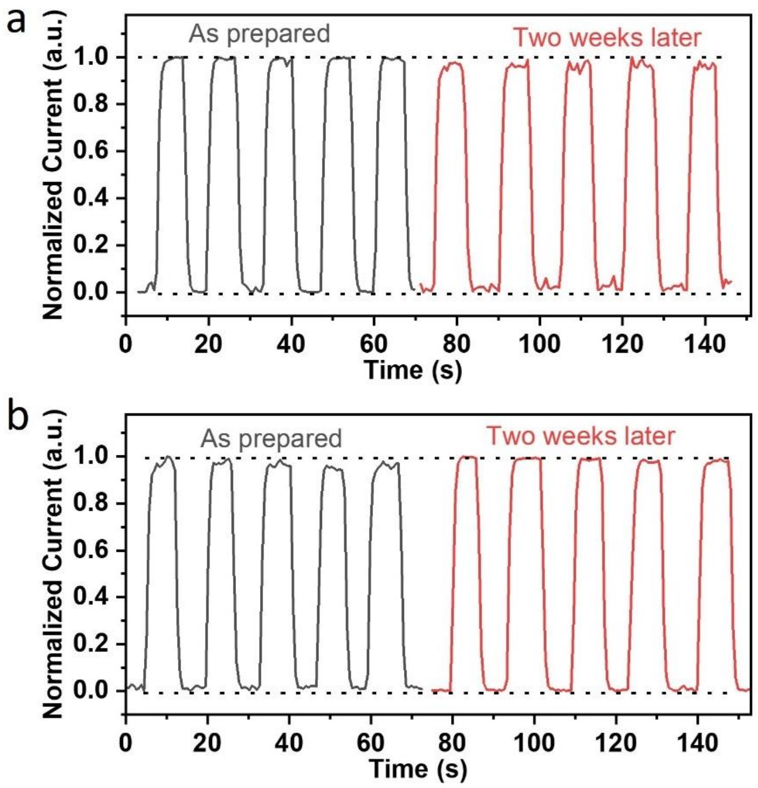

Figure S9. Time-dependent photocurrent of pristine a) and Pd-doped $Cs_2AgBiBr_6$ b) single-crystal photodetector devices after exposure to ambient conditions for two weeks. The excitation wavelengths for pristine and Pd-doped $Cs_2AgBiBr_6$ single-crystal photodetectors are 650 nm and 700 nm, respectively.

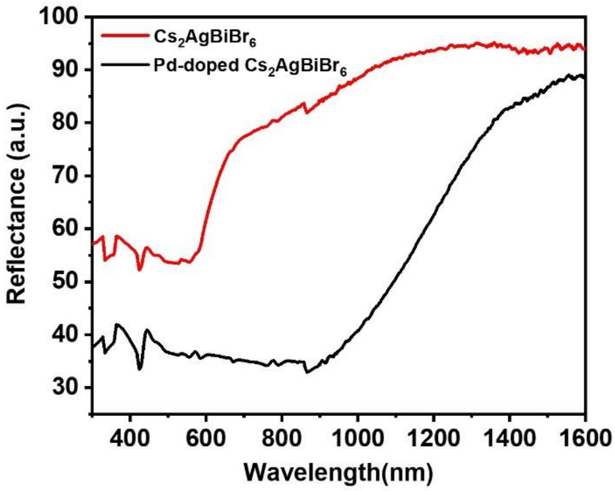

Figure S10. Diffuse reflectance spectra of pristine and Pd-doped $Cs_2AgBiBr_6$ crystal.

Table S1. ICP-OES spectrometry of pristine and Pd-doped $Cs_2AgBiBr_6$ crystals.

Sample*	Element	mass	unit	mol ratio
0.016%	Ag	137508.3	mg/kg	100%
	Bi	264201.2	mg/kg	99.17%
	Pd	18.6	mg/kg	0.014%
0.024%	Ag	121864.1	mg/kg	100%
	Bi	242048.3	mg/kg	102.52%
	Pd	19.3	mg/kg	0.016%
0.032%	Ag	119537.3	mg/kg	100%
	Bi	233105.4	mg/kg	100.66%
	Pd	21.1	mg/kg	0.018%
0.04%	Ag	115087.3	mg/kg	100%
	Bi	219250.6	mg/kg	98.33%
	Pd	21.6	mg/kg	0.019%
0.05%	Ag	118695.7	mg/kg	100%
	Bi	236615.4	mg/kg	102.89%
	Pd	88	mg/kg	0.075%
1%	Ag	74284.24	mg/kg	100%
	Bi	149514.7	mg/kg	103.90%
	Pd	791.4	mg/kg	1.10%
5%	Ag	96300.57	mg/kg	100%
	Bi	199920.1	mg/kg	107.20%
	Pd	2541.8	mg/kg	2.70%
10%	Ag	99631.7	mg/kg	100%
	Bi	211135.4	mg/kg	109.40%
	Pd	4564.4	mg/kg	4.60%
60%	Ag	93212.92	mg/kg	100%
	Bi	193344.2	mg/kg	107.10%
	Pd	5041.9	mg/kg	5.50%

*The ratio was calculated by comparing the mol ratio of $PdBr_2$ and $BiBr_3$ in the precursor. Further increasing the $PdBr_2$ ratio in precursor solution (> 60%) will not generate any crystals.

Table S2. Summary of the optical absorption range of metal halide perovskites.

Materials	Absorption range	Ref
$MAPbI_3$	400-780 nm (film); 400-820 nm (crystal)	[13,14]
$FAPbI_3$	400-870 nm (film); 400-900 nm (crystal)	[15,16]
$CsPbI_3$	400-720 nm (film); 350-760 nm (crystal)	[17,18]
$MASnI_3$	400-1015 nm (crystal)	[19]
$MASn_{0.48}Pb_{0.52}I_3$	400-1117 nm (crystal)	[19]
$FASnI_3$	400-905 nm (film)	[20]
$FASn_{0.5}Pb_{0.5}I_3$	500-1200 nm (film)	[21]
$CsSnI_3$	400-954 nm (film)	[22]
Cs_2SnI_6	354-765 nm (film)	[23]
Cs_2TiBr_6	400-689 nm (film)	[24]
$Cs_3Bi_2I_9$	350-600 nm (film); 300-633 nm (crystal)	[25,26]
$Cs_3Sb_2I_9$	354-605 nm (film); 500-640 nm (crystal)	[27,28]
$MA_2Au_2I_6$	500-1016 nm (film)	[29]
$Cs_2AgFeCl_6$	400-800 nm (crystal)	[30]
$Cs_2NaFeCl_6$	400-600 nm (crystal)	[31]
$Cs_2AgInCl_6$	400-443 nm (crystal)	[30]
$Cs_2AgTlBr_6$	400-1305 nm(crystal)	[32]
$Cs_2AgBiBr_6$	300-538 nm (film); 400-625 nm (red crystal); 400-715 nm (black crystal)	[26,33]
Cu-doped $Cs_2AgBiBr_6$	450-810 nm (crystal)	[34]
Sn-doped $Cs_2AgBiBr_6$	354-837 nm (crystal)	[35]
Sb-doped $Cs_2AgBiBr_6$	400-667 nm (crystal)	[36]
Fe-doped $Cs_2AgBiBr_6$	400-1350 nm (crystal)	[37]
Ru-doped $Cs_2AgBiBr_6$	400-1200 nm (crystal)	[38]
H-doped $Cs_2AgBiBr_6$	350-756 nm (film)	[39]
Pd-doped $Cs_2AgBiBr_6$	400-1400 nm (crystal)	This work

Table S3. Crystallographic data and refinement parameters for $Cs_2AgBiBr_6$ and Pd-doped $Cs_2AgBiBr_6$.

Formula	$Cs_2AgBiBr_6$	Pd-doped $Cs_2AgBiBr_6$
$D_{calc.}$/ g cm^{-3}	4,983	5,024
m/mm^{-1}	35,745	36,037
Formula Weight	1062,13	1062,13
Colour	red	black
Shape	block	block
Size/mm^3	0.20×0.15×0.10	0.20×0.15×0.10
T/K	150	150.15
Crystal System	cubic	cubic
Space Group	*Fm*-3*m*	*Fm*-3*m*
a/Å	11.2288(13)	11.1984(14)
b/Å	11.2288(13)	11.1984(14)
c/Å	11.2288(13)	11.1984(14)
a/°	90	90
b/°	90	90
g/°	90	90
V/Å^3	1415.8(5)	1404.3(5)
Z	4	4
Z'	0,020833	0,020833
Wavelength/Å	0,71073	0,71073
Radiation type	MoK_a	MoK_a
Q_{min}/°	3,142	3,151
Q_{max}/°	26,3	26,377
Measured Refl's.	3859	4051
Indep't Refl's	103	103
Refl's I≥2 *s*(I)	103	102
R_{int}	0,075	0,0724
Parameters	7	8
Restraints	0	0
Largest Peak	2,14	0,744
Deepest Hole	-3,383	-1,205
GooF	1,53	1,167
wR_2 (all data)	0,1131	0,0483
wR_2	0,1131	0,0482
R_1 (all data)	0,0496	0,0174
R_1	0,0496	0,0172

Table S4. The T_1(^{133}Cs) relaxation times determined for $Cs_2AgBiBr_6$ and Pd-doped $Cs_2AgBiBr_6$. The listed relaxation times and the corresponding standard deviations (*SD*) and residual sum of squares (*RSS*) were obtained by using single-component exponential functions.

Sample	T_1(^{133}Cs), s	*SD*	*RSS*
$Cs_2AgBiBr_6$	376	2.96e-3	1.4e-5
Pd-doped $Cs_2AgBiBr_6$	281	4.49e-3	3.6e-4

Table S5. The T_1(^{133}Cs) relaxation time was determined for $Cs_2AgBiBr_6$ and Pd-doped $Cs_2AgBiBr_6$ using the double-component exponential functions and the corresponding standard deviations (*SD*) and residual sum of squares (*RSS*).

Sample	Component #1		Component #2		*SD*	*RSS*
	T_1(^{133}Cs)$_I$, s	*I*, %	T_1(^{133}Cs)$_{II}$, s	*I*, %		
$Cs_2AgBiBr_6$ [a]	376	100	--	--	2.96e-3	1.4e-5
Pd-doped $Cs_2AgBiBr_6$ [b]	388±8	78±9	180±3	22±9	1.95±0.03e-3	6.8±0.6e-5

[a] No realistic second component #2 with the intensity larger than 1 % was found for the pristine $Cs_2AgBiBr_6$ system. [b] The component #1 was estimated and kept in the 400-300 s.

Table S6. Summary of the photo response region of photodetectors based on lead-based and lead-free perovskites.

	Device Structure	Photo response region	Reference
Lead-based	PEDOT:PSS/$MAPbI_{3-x}Cl_x$/PCBM	300-800 nm	[40]
	PEDOT:PSS/$MAPbI_3$/PCBM/C60	300-800 nm	[41]
	PTAA/$MAPbBr_3$/C60/BCP	350-570 nm	[42]
	PTAA/PEIE/$CsPbIBr_2$/PCBM/BCP	400-600 nm	[43]
	MoO_3/PEDOT:PSS/$CsPb_xSn_{1-x}I_3$/PC61BM	300–1100 nm	[44]
Lead-free	Au/$Cs_2AgBiBr_6$/Au	300-600 nm	[45]
	Au/(TMHD)$BiBr_5$ SC/Au	350-650 nm	[46]
	Au/$Cs_3Bi_2I_9$/Au	300-700 nm	[25]
	Au/$Cs_3Sb_2Br_9$/Au	420–500 nm	[47]
	Au/$Cs_3Cu_2I_5$/Au	200-320 nm	[48]
	Ag/$CsCu_2I_3$/Ag	300–700 nm	[49]
	Al/$MASnI_3$/Au	550–1000 nm	[50]
	Cr/Au/$FASnI_3$/Cr/Au	300-1000 nm	[51]
	FTO/Cs_2SnI_6/FTO	500-900 nm	[52]
	Au/$Cs_2AgInCl_6$/Au	340-400 nm	[53]
	SnO_2/$Cs_2AgBiBr_6$/TFB/Au	300-550 nm	[54]
	Au/Fe-doped $Cs_2AgBiBr_6$/Au	400-980 nm	[37]
	Au/Ru-doped $Cs_2AgBiBr_6$/Au	400-980 nm	[38]
	Au/Pd-doped $Cs_2AgBiBr_6$/Au	400-1300 nm	This Work

References:

[1] P. Kubelka, F. Munk, *Z. Tech. Phys.* **1931**, 12, 593– 601.
[2] H. Hamaed, M. W. Laschuk, V. V. Terskikh, R. W. Schurko, *J. Am. Chem. Soc.* **2009**, *131*, 8271–8279.
[3] J. Brus, *Solid State Nuclear Magnetic Resonance* **2000**, *16*, 151–160.
[4] P. E. Blöchl, *Phys. Rev. B* **1994**, *50*, 17953–17979.
[5] G. Kresse, J. Furthmüller, *Computational Materials Science* **1996**, *6*, 15–50.
[6] G. Kresse, J. Furthmüller, *Phys. Rev. B* **1996**, *54*, 11169–11186.
[7] G. Kresse, D. Joubert, *Phys. Rev. B* **1999**, *59*, 1758–1775.
[8] J. P. Perdew, A. Ruzsinszky, G. I. Csonka, O. A. Vydrov, G. E. Scuseria, L. A. Constantin, X. Zhou, K. Burke, *Phys. Rev. Lett.* **2008**, *100*, 136406.
[9] A. V. Krukau, O. A. Vydrov, A. F. Izmaylov, G. E. Scuseria, *The Journal of Chemical Physics* **2006**, *125*, 224106.
[10] A. Zunger, S.-H. Wei, L. G. Ferreira, J. E. Bernard, *Phys. Rev. Lett.* **1990**, *65*, 353–356.
[11] J. Buckeridge, D. O. Scanlon, A. Walsh, C. R. A. Catlow, *Computer Physics Communications* **2014**, *185*, 330–338.
[12] C. Freysoldt, J. Neugebauer, C. G. Van De Walle, *Phys. Rev. Lett.* **2009**, *102*, 016402.
[13] F. Ji, S. Pang, L. Zhang, Y. Zong, G. Cui, N. P. Padture, Y. Zhou, *ACS Energy Lett.* **2017**, *2*, 2727–2733.
[14] M. I. Saidaminov, A. L. Abdelhady, B. Murali, E. Alarousu, V. M. Burlakov, W. Peng, I. Dursun, L. Wang, Y. He, G. Maculan, A. Goriely, T. Wu, O. F. Mohammed, O. M. Bakr, *Nat Commun* **2015**, *6*, 7586.
[15] S. Pang, H. Hu, J. Zhang, S. Lv, Y. Yu, F. Wei, T. Qin, H. Xu, Z. Liu, G. Cui, *Chem. Mater.* **2014**, *26*, 1485–1491.
[16] M. I. Saidaminov, A. L. Abdelhady, G. Maculan, O. M. Bakr, *Chem. Commun.* **2015**, *51*, 17658–17661.
[17] N.-K. Cho, H.-J. Na, J. Yoo, Y. S. Kim, *Commun Mater* **2021**, *2*, 30.
[18] D. B. Straus, S. Guo, R. J. Cava, *J. Am. Chem. Soc.* **2019**, *141*, 11435–11439.
[19] C. C. Stoumpos, C. D. Malliakas, M. G. Kanatzidis, *Inorg. Chem.* **2013**, *52*, 9019–9038.
[20] X. Liu, Y. Wang, F. Xie, X. Yang, L. Han, *ACS Energy Lett.* **2018**, *3*, 1116–1121.
[21] S. Shao, Y. Cui, H. Duim, X. Qiu, J. Dong, G. H. Ten Brink, G. Portale, R. C. Chiechi, S. Zhang, J. Hou, M. A. Loi, *Advanced Materials* **2018**, *30*, 1803703.
[22] M. H. Kumar, S. Dharani, W. L. Leong, P. P. Boix, R. R. Prabhakar, T. Baikie, C. Shi, H. Ding, R. Ramesh, M. Asta, M. Graetzel, S. G. Mhaisalkar, N. Mathews, *Advanced Materials* **2014**, *26*, 7122–7127.
[23] B. Saparov, J.-P. Sun, W. Meng, Z. Xiao, H.-S. Duan, O. Gunawan, D. Shin, I. G. Hill, Y. Yan, D. B. Mitzi, *Chem. Mater.* **2016**, *28*, 2315–2322.
[24] M. Chen, M.-G. Ju, A. D. Carl, Y. Zong, R. L. Grimm, J. Gu, X. C. Zeng, Y. Zhou, N. P. Padture, *Joule* **2018**, *2*, 558–570.
[25] Y. Zhang, Y. Liu, Z. Xu, H. Ye, Z. Yang, J. You, M. Liu, Y. He, M. G. Kanatzidis, S. Liu, *Nat Commun* **2020**, *11*, 2304.
[26] F. Ji, B. Zhang, W. M. Chen, I. A. Buyanova, F. Wang, G. Boschloo, *Advanced Science* **2023**, 2306391.
[27] B. Saparov, F. Hong, J.-P. Sun, H.-S. Duan, W. Meng, S. Cameron, I. G. Hill, Y. Yan, D. B. Mitzi, *Chem. Mater.* **2015**, *27*, 5622–5632.
[28] A. Singh, S. Satapathi, *Advanced Optical Materials* **2021**, *9*, 2101062.
[29] B. Ghosh, B. Febriansyah, P. C. Harikesh, T. M. Koh, S. Hadke, L. H. Wong, J. England, S. G. Mhaisalkar, N. Mathews, *Chem. Mater.* **2020**, *32*, 6318–6325.

[30] F. Ji, F. Wang, L. Kobera, S. Abbrent, J. Brus, W. Ning, F. Gao, *Chem. Sci.* **2021**, *12*, 1730–1735.
[31] F. Ji, J. Klarbring, B. Zhang, F. Wang, L. Wang, X. Miao, W. Ning, M. Zhang, X. Cai, B. Bakhit, M. Magnuson, X. Ren, L. Sun, M. Fahlman, I. A. Buyanova, W. M. Chen, S. I. Simak, I. A. Abrikosov, F. Gao, *Advanced Optical Materials* **2023**, 2301102.
[32] A. H. Slavney, L. Leppert, A. Saldivar Valdes, D. Bartesaghi, T. J. Savenije, J. B. Neaton, H. I. Karunadasa, *Angew. Chem. Int. Ed.* **2018**, *57*, 12765–12770.
[33] F. Ji, J. Klarbring, F. Wang, W. Ning, L. Wang, C. Yin, J. S. M. Figueroa, C. K. Christensen, M. Etter, T. Ederth, L. Sun, S. I. Simak, I. A. Abrikosov, F. Gao, *Angew. Chem.* **2020**, *132*, 15303–15306.
[34] F. Ji, Y. Huang, F. Wang, L. Kobera, F. Xie, J. Klarbring, S. Abbrent, J. Brus, C. Yin, S. I. Simak, I. A. Abrikosov, I. A. Buyanova, W. M. Chen, F. Gao, *Adv. Funct. Mater.* **2020**, *30*, 2005521.
[35] K. P. Lindquist, S. A. Mack, A. H. Slavney, L. Leppert, A. Gold-Parker, J. F. Stebbins, A. Salleo, M. F. Toney, J. B. Neaton, H. I. Karunadasa, *Chem. Sci.* **2019**, *10*, 10620–10628.
[36] K. Du, W. Meng, X. Wang, Y. Yan, D. B. Mitzi, *Angew. Chem. Int. Ed.* **2017**, *56*, 8158–8162.
[37] G. Liu, Z. Zhang, C. Wu, Y. Zhang, X. Li, W. Yu, G. Yao, S. Liu, J. Shi, K. Liu, Z. Chen, L. Xiao, B. Qu, *Adv Funct Materials* **2022**, *32*, 2109891.
[38] Z. Zhang, G. Liu, W. Guo, X. Li, Y. Zhang, C. Wu, B. Qu, J. Shi, Z. Chen, L. Xiao, *Mater. Adv.* **2022**, *3*, 4932–4937.
[39] Z. Zhang, Q. Sun, Y. Lu, F. Lu, X. Mu, S.-H. Wei, M. Sui, *Nat Commun* **2022**, *13*, 3397.
[40] L. Dou, Y. Yang, J. You, Z. Hong, W.-H. Chang, G. Li, Y. Yang, *Nat Commun* **2014**, *5*, 5404.
[41] Q. Lin, A. Armin, D. M. Lyons, P. L. Burn, P. Meredith, *Advanced Materials* **2015**, *27*, 2060–2064.
[42] C. Bao, Z. Chen, Y. Fang, H. Wei, Y. Deng, X. Xiao, L. Li, J. Huang, *Advanced Materials* **2017**, *29*, 1703209.
[43] C. Bao, J. Yang, S. Bai, W. Xu, Z. Yan, Q. Xu, J. Liu, W. Zhang, F. Gao, *Adv. Mater.* **2018**, *30*, 1803422.
[44] H. L. Zhu, H. Lin, Z. Song, Z. Wang, F. Ye, H. Zhang, W.-J. Yin, Y. Yan, W. C. H. Choy, *ACS Nano* **2019**, *13*, 11800–11808.
[45] L.-Z. Lei, Z.-F. Shi, Y. Li, Z.-Z. Ma, F. Zhang, T.-T. Xu, Y.-T. Tian, D. Wu, X.-J. Li, G.-T. Du, *J. Mater. Chem. C* **2018**, *6*, 7982–7988.
[46] C. Ji, P. Wang, Z. Wu, Z. Sun, L. Li, J. Zhang, W. Hu, M. Hong, J. Luo, *Adv Funct Materials* **2018**, *28*, 1705467.
[47] P. Liu, Y. Liu, S. Zhang, J. Li, C. Wang, C. Zhao, P. Nie, Y. Dong, X. Zhang, S. Zhao, G. Wei, *Advanced Optical Materials* **2020**, *8*, 2001072.
[48] W.-Q. Liang, Y. Li, J.-L. Ma, Y. Wang, J.-J. Yan, X. Chen, D. Wu, Y.-T. Tian, X.-J. Li, Z.-F. Shi, *Nanoscale* **2020**, *12*, 17213–17221.
[49] Z. Li, Z. Li, Z. Shi, X. Fang, *Adv Funct Materials* **2020**, *30*, 2002634.
[50] A. Waleed, M. M. Tavakoli, L. Gu, Z. Wang, D. Zhang, A. Manikandan, Q. Zhang, R. Zhang, Y.-L. Chueh, Z. Fan, *Nano Lett.* **2017**, *17*, 523–530.
[51] C. Liu, Q. Tai, N. Wang, G. Tang, H. Loi, F. Yan, *Advanced Science* **2019**, *6*, 1900751.
[52] X. Han, J. Liang, J. Yang, K. Soni, Q. Fang, W. Wang, J. Zhang, S. Jia, A. A. Martí, Y. Zhao, J. Lou, *Small* **2019**, *15*, 1901650.

[53] J. Luo, S. Li, H. Wu, Y. Zhou, Y. Li, J. Liu, J. Li, K. Li, F. Yi, G. Niu, J. Tang, *ACS Photonics* **2018**, *5*, 398–405.
[54] J. Yang, C. Bao, W. Ning, B. Wu, F. Ji, Z. Yan, Y. Tao, J. Liu, T. C. Sum, S. Bai, J. Wang, W. Huang, W. Zhang, F. Gao, *Advanced Optical Materials* **2019**, 1801732.
